# Assessing the association between overcrowding and human physiological stress response in different urban contexts: a case study in Salzburg, Austria

**DOI:** 10.1186/s12942-023-00334-7

**Published:** 2023-06-21

**Authors:** Zhaoxi Zhang, Kristýna Měchurová, Bernd Resch, Prince Amegbor, Clive E. Sabel

**Affiliations:** 1 Department of Environmental Science, Aarhus University, 4000 Roskilde, Denmark; 2 Spatial Services GmbH, 5020 Salzburg, Austria; 3 Department of Geoinformatics, University of Salzburg, 5020 Salzburg, Austria; 4 Center for Geographic Analysis, Harvard University, Cambridge, MA 02138 USA; 5 School of Global Public Health, New York University, New York, 10003 USA; 6 Department of Public Health, Aarhus University, 8000 Aarhus, Denmark; 7 BERTHA, The Danish Big Data Centre for Environment and Health, Aarhus University, 8000 Aarhus, Denmark

**Keywords:** Wearable device, Machine learning, Individual-level measurement, Public open space

## Abstract

Overcrowding in densely populated urban areas is increasingly becoming an issue for mental health disorders. Yet, only few studies have examined the association between overcrowding in cities and physiological stress responses. Thus, this study employed wearable sensors (a wearable camera, an Empatica E4 wristband and a smartphone-based GPS) to assess the association between overcrowding and human physiological stress response in four types of urban contexts (green space, transit space, commercial space, and blue space). A case study with 26 participants was conducted in Salzburg, Austria. We used Mask R-CNN to detect elements related to overcrowding such as human crowds, sitting facilities, vehicles and bikes from first-person video data collected by wearable cameras, and calculated a change score (CS) to assess human physiological stress response based on galvanic skin response (GSR) and skin temperature from the physiological data collected by the wristband, then this study used statistical and spatial analysis to assess the association between the change score and the above elements. The results demonstrate the feasibility of using sensor-based measurement and quantitative analysis to investigate the relationship between human stress and overcrowding in relation to different urban elements. The findings of this study indicate the importance of considering human crowds, sitting facilities, vehicles and bikes to assess the impact of overcrowding on human stress at street level.

**Supplementary Information:**

The online version contains supplementary material available at 10.1186/s12942-023-00334-7.

## Introduction

Global increases in population and housing density have exacerbated overcrowding in cities, which has been associated with feelings of negative emotional arousal [1] and a higher risk of mental health disorders [2–7]. Although people’s perceived violation of their personal space in crowded places can increase their stress levels [8, 9], there are a limited number of studies that have examined the relationships between overcrowding in the urban environment and physiological stress response at the individual level. Due to the lack of practical tools and high-resolution data, previous research to date has been severely limited in two ways. First, previous studies have often focused on ‘macro’ urban characteristics, such as density and clusters of overcrowding in cities, with few studies examining the impact of ‘micro’ characteristics of public open space, such as street-level measurements of overcrowding and its impact on human health. Second, few studies focus on the overcrowding in different types of urban contexts to discuss the complexity of urban characteristics and their impact on people’s mental health. Therefore, successful methods to accurately measure human physiological stress response in urban environments are needed. For example, novel wearable devices can monitor people’s experience, environmental exposures and real-time physiological responses outside the laboratory.

Taking advantage of personal tracking techniques, the focus of this paper is twofold: (1) to test the feasibility of using a wearable camera and machine learning to assess overcrowding at street level, and integrating image data with human physiological response measured by a health tracker in the urban environment, and (2) to assess the relationship between urban elements that may increase overcrowding and human physiological stress response at the individual level.

## Related work

### Overcrowding in the built environment

Since the mid-1960s, many urban planning studies have been focused on discovering the health effects of the high population and housing density in urban areas and humans’ perception of overcrowding [1, 7, 10, 11]. Recent researchers have focused on the social sustainability [12], traffic problems[13], air quality [14] and extreme heat [15] in higher density area, which are  closely related to human wellbeing. Previous research in social psychology has identified the difference between “density” and “crowding”: “density” in terms of spatial parameters and describes the physical condition objectively, but “crowding” refers to the individual’ s perception of spatial restriction caused by the interaction of spatial, social, and personal factors [16–18]. Previous studies have mentioned that overcrowding caused by pedestrians, cars and bicycles can negatively influence human’s travel choices [19], physical activities [2, 7] and mental health [5] in cities, and have explored how to mitigate the negative effects of overcrowding, such as increasing urban greenery [20].

Based on this, SC Choi, A Mirjafari and HB Weaver [16] postulated that ‘number of people per unit’, ‘physical environment factors’, ‘personal factors’ and ‘ways of adaptation to crowding’ are factors that affect overcrowding. Many researchers have adopted ‘number of people in a given space’ to quantify the level of crowding [11, 21]. In terms of the crowdedness spot in the urban environment, it is a crowded area with an abnormal number of objects, such as pedestrians, parked bicycles, moving vehicles, or potential points of interest, such as exhibitions and commercial promotions [22–24].

Personal space in crowded environments is directly related to an individual’s awareness of spatial restriction, which might cause the experience of stress [17]. Over the last half century, the concept of proxemics—the study of interpersonal space and spatial distances in different situations [8]—and personal space have been conceived as a possible explanation for the stressful effects of crowding. Some studies have shown that a reasonable personal distance^1^ is close to “arm’s length without touching distance” [8, 25, 26], and that the violation of human’s expectations in a crowded built environment can affect comfort, perception of crowding and human spatial behaviour [9, 26–29], which may generate negative effects on psychological responses [30, 31]. Based on the concept of personal space, recent studies have also explored the possible shape of personal space [32] and simulated pedestrian crowd dynamics [27, 33–35].

Gehl has stated that urban design should respect personal space and ensure personal distance in the built environment [30]. Particularly since the COVID-19 pandemic, studies have clearly highlighted the importance of personal space in cities and suggested that the public sector should find solutions to overcome the pedestrian congestion [36–40]. There is a need to explore the street-level measurements to better understand the impact of overcrowding on human health.

### Estimation of crowd size

Compared to traditional methods, such as field observations, mapping, and interviews [1, 3, 7, 9, 11, 16, 30, 31], researchers have recently explored modern technologies and devices to automatically count people’s gatherings in a given space. For example, Kanjo et al. [41] have used Wi-Fi probing and density-based clustering techniques to detect people’s gatherings; Booranawong et al. [42] have used wireless networks to locate people by monitoring the changes or strength of radio signals; Ghose et al. [43] have detected proximity and crowdedness by using Bluetooth and mobile phones at public places. Although these new devices reduce the cost of time and labour, these methods can only count people who turn on the device (e.g. GPS or Bluetooth on the smartphones), while the radio signal is limited by the area coverage and cost.

There are many novel applications of imagery in urban studies, such as vision-based traffic monitoring systems and surveillance systems [44, 45], Google Street View (GSV) [46–48] and remote sensing (RS) imagery [49]. Some studies have applied deep learning methods, such as CNNs (Convolutional Neural Networks), Faster R-CNNs, Mask RCNNs, YOLO (You Only Look Once) algorithm and SegNet (Semantic Segmentation Model) to automatically detect objects such as “person”, “car”, “tree” and “bicycle” [50–52] from images. In order to better capture the contextual information, studies have shown the possibility of employing cameras in the urban environment. For example, fixed infrared cameras have been used for passive collection of imagery about traffic flow near a road crossing [53], street parking [54] and people visiting a playground [55]. Moreover, portable cameras, such as wearable cameras, are widely used to track individuals’ experiences at street level [56, 57] and are combined with GPS as a package to record views while moving [58, 59]. In the same vein, Kelly et al. [60] have emphasised the importance of protecting the confidentiality of participants and third parties while using wearable cameras.

The use of wearable cameras for urban studies is still in its infancy. However, we believe that wearable cameras offer a novel way of measuring street-level overcrowding from an individual’s perspective, as they capture personal images from footpaths that represent personal space.

### Health trackers and stress assessment

Wearable health trackers have been employed to monitor the human body’s physiological responses in the urban environment [61]. Recent studies have combined these wearable health trackers with other environmental monitors carried by individuals for the aim of studying the relationship between health effects and exposure to environmental stressors (e.g., noise, air pollutant, temperature) [62–67]. Zhang et al. [68] stated the most commonly used health tracker for integration is the wristband, such as Empatica E4^2^, Microsoft Band 2^3^, and Fitbit^4^.

Previous studies have used heart rate (HR), heart rate variability (HRV), skin temperature (ST) and galvanic skin response (GSR) to assess people’s emotional responses. Researchers have found that human stress is associated with an increase in GSR [69] and a decrease in finger temperature [70]. GSR, also known as skin conductance or electrodermal activity (EDA), is often used to detect stress patterns [71, 72]. An increasing number of urban studies employ wristbands and GPS devices to measure people’s stress responses while walking or cycling and then map out the hotspots of stress in the built environment [62, 73].

Although recent urban studies have used health trackers and urban imagery (e.g., Google Street View) to investigate the effects of urban features on health [74–76], Zhang et al. [77] have demonstrated the benefits of integrating wearable cameras and health trackers for street-level research. Focusing on overcrowding, this study aims to take advantage of wearable cameras, machine learning, statistical and spatial analysis to assess the personal exposure to overcrowding in urban space.

## Methods

### Data collection

The fieldwork presented in this paper was conducted in Salzburg, Austria, from November to December 2021. The study was carried out in accordance with the Declaration of Helsinki and complies with the EU’s General Data Protection Regulation (GDPR) legislation. In this study, 26 participants were recruited via university networks and social media (16 females, 10 males), with the average age being 28 years (aged between 18 and 54 years). After the fieldwork, participants were thanked for their time and effort and given a small gift card. Due to language requirements, recruitment was limited to English speakers.

**Fig. 1:**
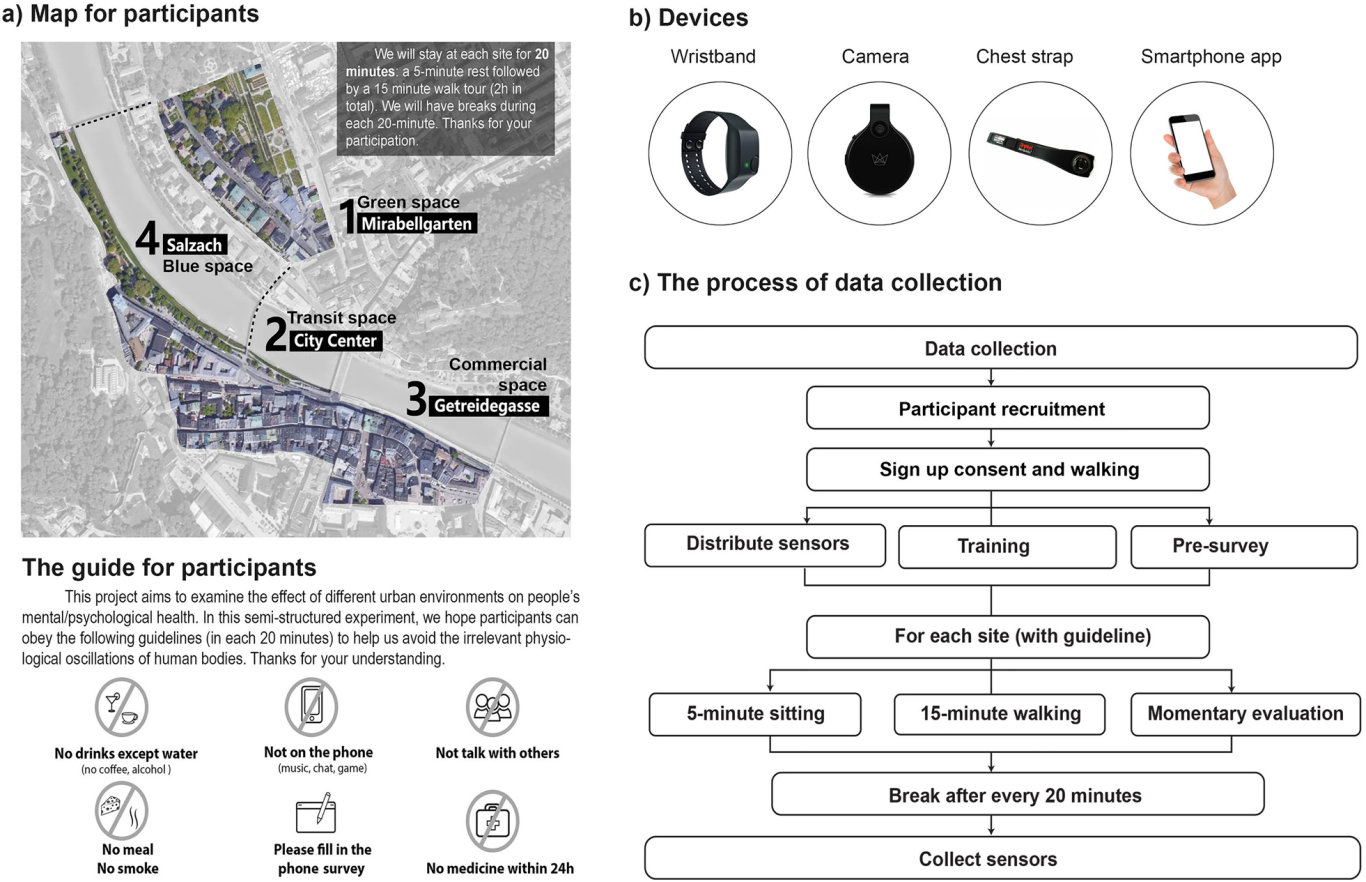
Map, devices, participant guide and data collection process

Participants were equipped with (1) a wearable camera, “FrontRow (FR)”, to record first-person videos of the front view during walking; (2) a wearable sensor, “Empatica E4”, to collect skin conductance and skin temperature; (3) smartphone with the “eDiary” smartphone application developed by PLUS [78] to collect participants’ momentary psychological feedback by asking participants to enter their subjective observations, i.e., when and where they felt a particular emotion, together with the cause of that feeling (Fig. 1b). The effectiveness of using the “eDiary” application to control E4 and store data has been demonstrated in previous work [75, 78]. Participants were also provided with the “Zephyr Bioharness 3” chest strap to record electrocardiogram (ECG) data. The eDiary app connected the E4 and Bioharness sensors via Bluetooth to store data, and automatically linked up the physiological data with the phone's built-in GPS based on the timestamps. At the same time, the videos were stored independently in the FR cameras. We extracted a single image frame every second and merged the images with data from the eDiary using timestamps. This study mainly focused on E4 data and images to explore the association between stress responses and overcrowding in personal space.

The study area was located in the centre of Salzburg and consisted of four sites: Mirabellgarten (green space), city centre (transit space), Getreidegasse (commercial space) and Salzach (blue space). There was a maximum of five participants at each slot. We provided participants with a printed map (Fig. 1a) and led them on a walk from site 1 to site 4. Participants spent 20 minutes at each site: 5-minute sitting followed by 15-minute walking (Fig. 1c), and each participant walked independently. In order to avoid irrelevant physiological oscillations of the human body, participants were asked to obey the following guidelines at each site:

- No drinks (e.g., coffee, alcohol) 1 hour before the walk and throughout the walk; we provided water during the walk.
- No smoking during the walk.
- No eating, but we provided bananas during the break and sandwiches after the walk.
- No use of phone (e.g., music, chats and games) during every 20-minute period.
- No talking with others during every 20-minute period.
- No medicine 24 hours before the walk.

We provided three available sessions for participants to register within each day, 9am-12pm, 1pm-4pm, and 5pm-8pm. However, since this data collection was conducted in the winter, the camera performed poorly at night and the battery quickly ran out in the low winter temperatures. Therefore, we only included data collected during the daytime. The smartphone application sometimes had connection problems and loading errors; therefore, we included the data from 20 participants after excluding missing data.

### Assessment of physiological stress response

The stress detection was implemented based on the assumption that skin conductance increases, and skin temperature decreases when a negative experience occurs [69, 70]. Based on this, Kyriakou and Resch (2019) defined five rules, including R1–GSR amplitude increase; R2–Skin temperature decrease; R3–GSR rising time; R4– GSR response slope, R5–Duration, to detect moments of stress (MOS) from wearable physiological sensors [74, 75]. Since this study focused more on the changes in physiological stress response during people’s movement rather than any specific stress event, so R1 to R4 were used to evaluate the continuous and momentary changes in human physiological stress response, and we called it change score (CS).

**Fig. 2:**
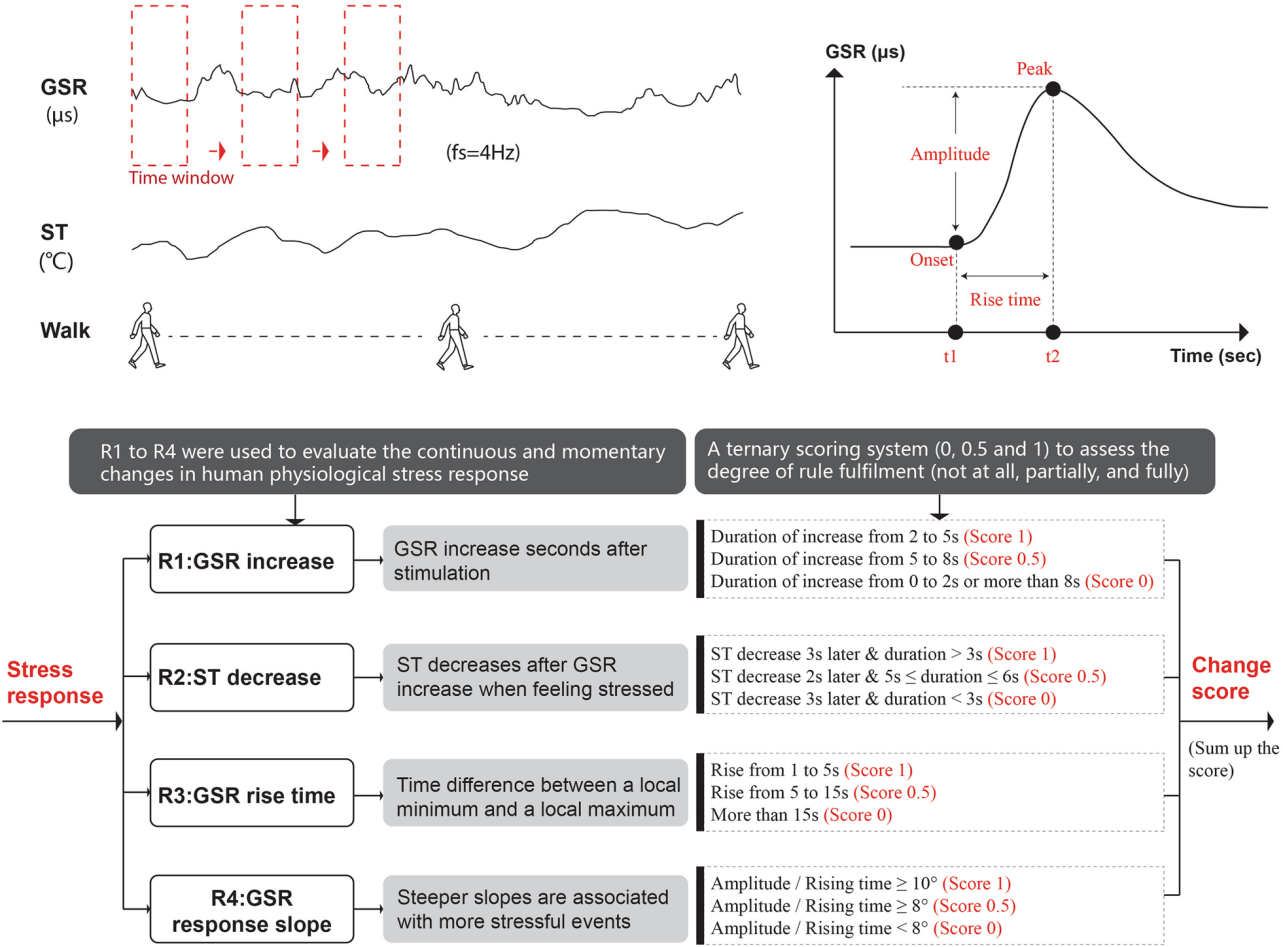
Assessment of physiological stress response based on GSR and ST

First, we filtered the GSR and ST by the pre-processing rules used by Kyriakou and Resch (2019) to maintain data with the correct frequency. Second, we adopted the ternary scoring system (0, 0.5, 1) from Kyriakou and Resch (2019) to assess the degree of rule fulfilment: score 1 (complete), score 0.5 (partial) and score 0 (no fulfilment), as shown in Fig. 2. Afterwards, the change score for a second is calculated by the sum of sc ∗ , where sc is the given score for the rules and wn is the associated weight of the rules [74, 75]. The CS is continuous at every second and ranges from zero to 80, provided that all rules are scored with “1”. Therefore, a lower change score indicates a weak physiological stress response, while a higher change score, in contrast, indicates a strong physiological stress response.

### Image detection

**Fig. 3:**
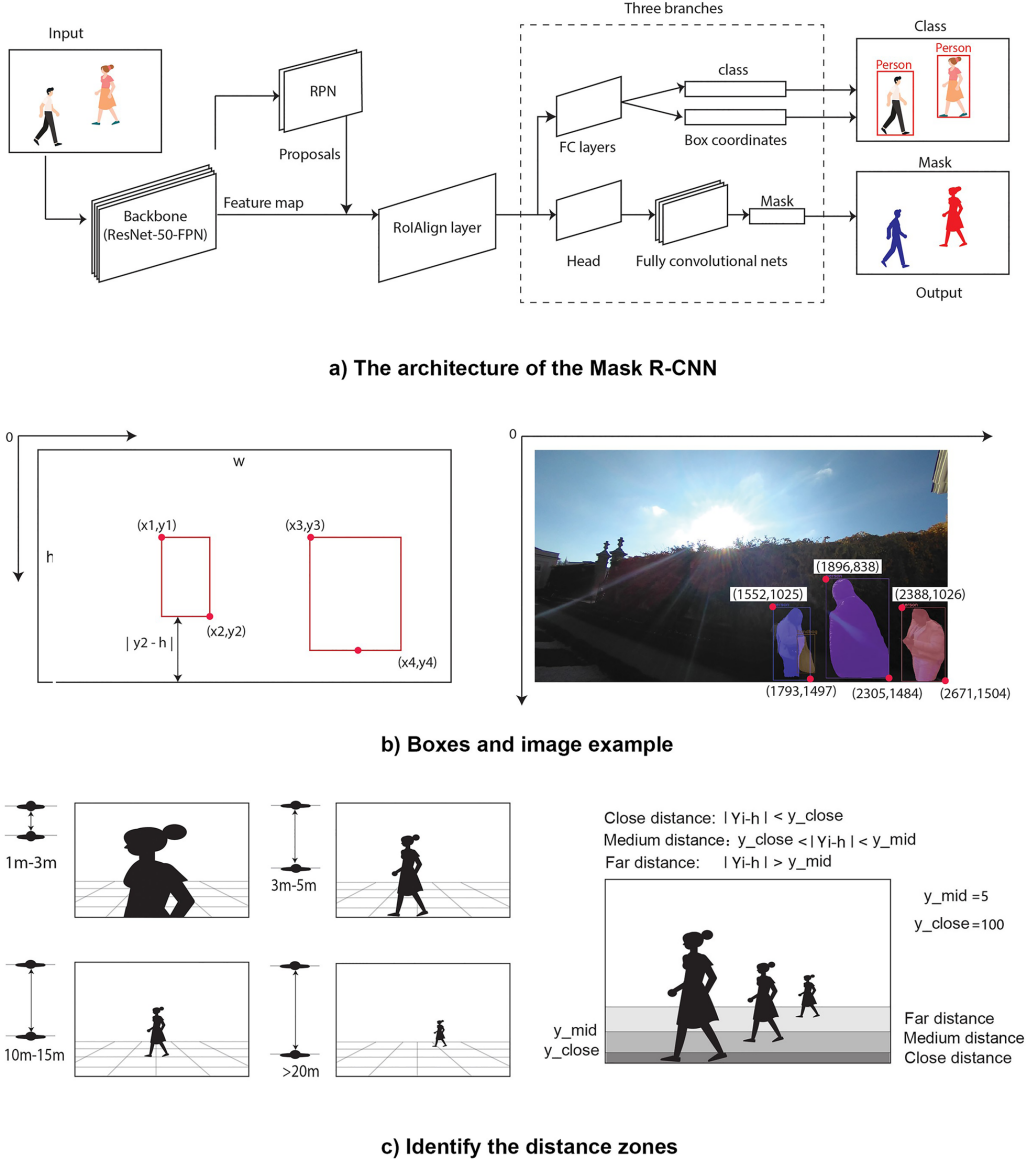
Classifying human crowds from imagery into different distance zones

In this study, Mask R-CNN, a region-based convolutional neural network [79], was adopted to automatically identify elements from the imagery (Fig. 3a). Mask R-CNN can efficiently detect objects and simultaneously generate an instance segmentation mask for each object (Fig. 3b). We used the Microsoft COCO (Common Objects in Context) training set to train the Mask R-CNN model and generated the coordinates of the box, the class of layer, the score of the prediction and the instance segmentation mask. From Mask R-CNN model, this paper detect the number of : “person”, “car”, “bicycle”, “motorcycle”, “bus”, “truck”, “chair”, “bench”, “dining table”, and classify these objects into four catogries related to overcrowding: human crowds (“person”), motor vehicles (“bus”, “car”, “truck”, “motorcycle”), bikes (“bicycle”) and sitting facilities (chair”, “bench”, “dining table”).

Based on the result of Mask RCNN, this study further assessed the proxemics in the human crowds by calculating the distance of the boxes labelled ‘person’ to the ground of the image (Fig. 3c) and categorising the human crowds into four distance zones: ‘personal space’, ‘close distance’, ‘medium distance’ and ‘far distance’. According to the principle of perspective, people at a distance appear small and those close to the observer appear large. Persons who are close to the observer are also closer to the bottom of the image. Therefore, we calculated the ratio of each mask from Mask RCNN and the distance from the bottom line to the ground of the image, which is |Yi - h|. To determine the distance zones of each object, we randomly selected a small number of images to find out the average number of |Yi - h|. We then decided on the location of two lines, y_close and y_mid, to separate the area of ‘close distance’, ‘medium distance’ and ‘far distance’. If the |Yi - h| of a box falls in the (5,100), it means that the ‘person’ is in the middle distance from the observer. If the |Yi - h| of a box is over 100, it means that the ‘person’ is in the far distance from the observer. Otherwise, it is in the close distance from the observer. Sometimes, the ‘person’ in the image is comparably enormous to others, and the possible reason is that the crowd has invaded the observer’s personal space. Therefore, if the proportion of each mask from Mask RCNN is greater than 10%, it is in the category of ‘personal space’.

### Analysis

To address the first objective, a generalised linear mixed model (GLMM) was used to examine the association between exposure to human crowds and human physiological stress response, as GLMMs can take into account the non-independent variables as random effect parameters for the correlation between observations, and deal with the hierarchical structure of data nested within time slots and participants. We used the detected persons (the human crowd) in four distance zones (‘personal space’, ‘close distance’, ‘medium distance’ and ‘far distance’) and the number of motor vehicles, bikes and sitting facilities detected from the images as predictors and the change score as the dependent variable to explore how exposure to human crowds was significantly associated with physiological stress responses in different urban contexts.

As the results of the GLMM analysis only indicate the global relationship between predictors and responses, local spatial variation in the association between predictors and the change score is not explored thoroughly (Additional file 2: Appendix Table S1). Geographically weighted regression (GWR) within a GIS platform was used to estimate local spatial variation in the association between the predictors and response (Additional file 1: Appendix Fig. S1). We used the number of sitting facilities, vehicles and bikes as predictors and change score as response to compare the variation in coefficients between urban elements and the change score by location.

## Results

### GLMM analysis: stress and overcrowding

As shown in Table 1, the change score of physiological stress is statistically positively associated with the human crowds in the personal space in the commercial space. In contrast, the result shows a negative association between the human crowds in the close distance and the change score in the green space and the transit space. Furthermore, the number of bikes is positively correlated with the change score in the commercial space, while the number of motor vehicles is negatively correlated with the change score in the transit space. However, GLMMs are not sufficient to provide an understanding of the results, as they only provide the global perspective of the analysis.

**Table 1:** GLMM result between the number of detected objects and change score (data point)

Variable	Green space	Transit space	Commercial space	Blue space
	Estimates (95% CI)	Estimates (95% CI)	Estimates (95% CI)	Estimates (95% CI)
Human crowds in the personal space	− 2.02 (− 4.62 to 0.59)	0.91 (− 1.79 to 3.61)	3.85 (2.05 to 5.66)***	1.11 (− 1.57 to 3.79)
Human crowds in the close distance	− 0.97 (1.45 to − 0.49)***	− 0.37 (− 0.59 to − 0.15)***	− 0.02 (− 0.23 to 0.20)	0.15 (− 0.20 to 0.50)
Human crowds in the medium distance	− 0.06 (− 0.30 to 0.18)	0.04 (− 0.10 to 0.17)	− 0.08 (− 0.20 to 0.04)	− 0.29 (− 0.50 to − 0.07)**
Human crowds in the far distance	− 0.23 (− 0.35 to − 0.11)***	− 0.12 (− 0.19 to − 0.06)***	0.00 (− 0.05 to 0.05)	− 0.06 (− 0.16 to 0.04)
Motor vehicles	1.70 (− 0.80 to 4.20)	− 0.25 (− 0.36 to − 0.13)***	− 0.03 (− 0.17 to 0.11)	0.12 (− 0.28 to 0.04)
Bikes	0.20 (− 1.16 to 1.55)	− 0.09 (− 0.30 to 0.12)	0.23 (0.07 to 0.39)**	− 0.31 (− 0.57 to − 0.05)*
sitting facilities	0.36 (− 0.17 to 0.88)	− 0.19 (− 0.78 to 0.41)	0.39 (− 0.02 to 0.80)	− 0.46 (− 1.03 to 0.10)
Random effects				
σ2	263.67	269.91	269.92	269.41
τ00 time_slot	0.00	0.00	0.00	0.00
τ00 person_ID	28.28	25.63	37.30	15.88
τ00 gender	0.00	0.00	0.00	0.00
ICC	0.10	–	–	0.06
N timeslot	2	2	2	2
N person_ID	19	23	21	17
N gender	2	2	2	2
Observation	16,082	26,340	32,184	23,402
Marginal R2/conditional R2	0.002/0.099	0.002/NA	0.001/NA	0.001/0.057

(1) ***Significant < 0.1%, **significant < 1% level, *significant < 5% level. (2) ‘N person_ID’ means ‘the number of participants’, as each participant walked from site 1 to site 4 each time. ‘N timeslot’ means the time sessions, we include data collected from 9 am–12 pm and 1 pm–4 pm. (3) The analysis is based on the fused data points collected from three sensors rather than individuals. The data points were separated into different locations by the GPS locations.

### GWR analysis

**Fig. 4:**
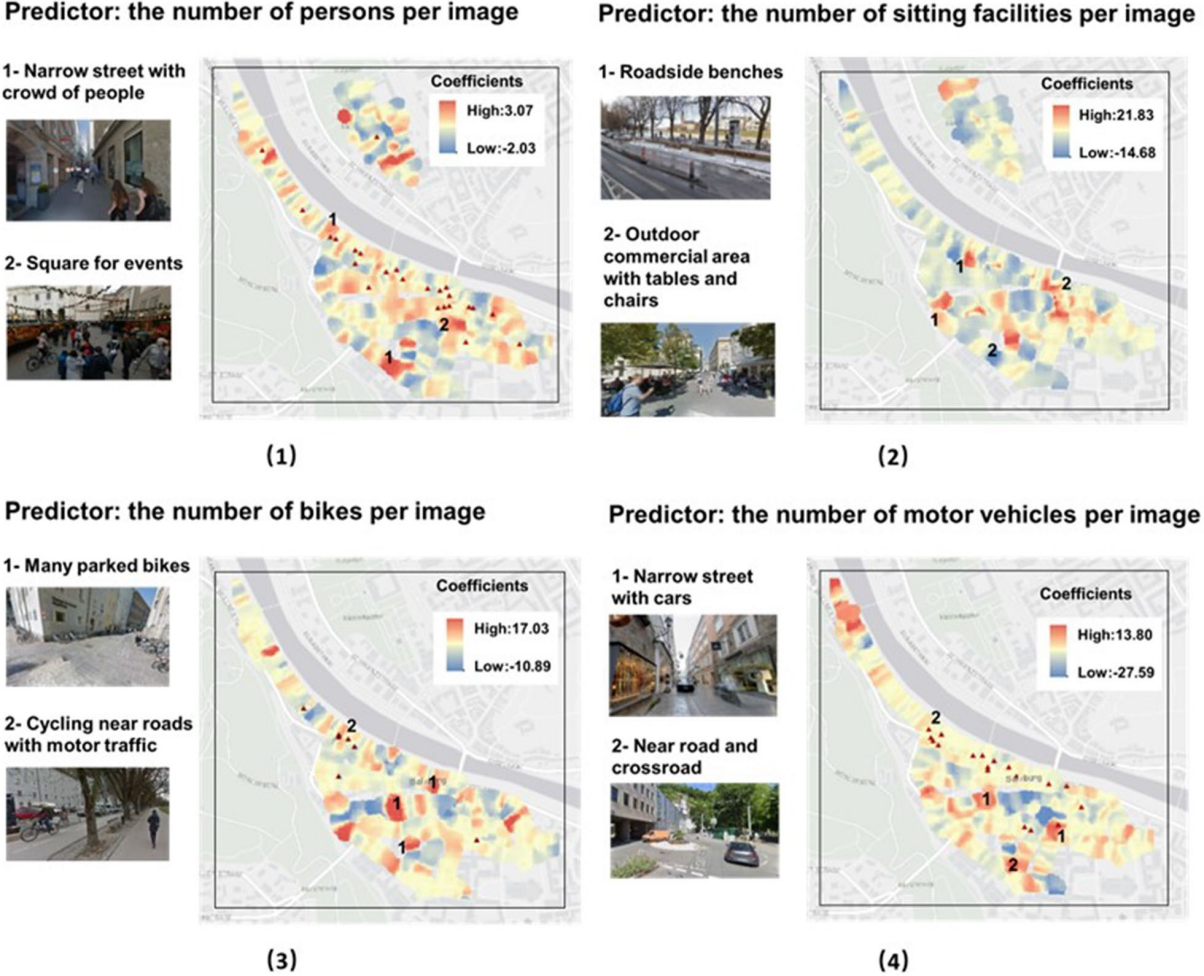
GWR analysis of stress responses in Salzburg. The ▲ on the map means the locations where participants reported their negative feeling due to the predictor. For example, on the GWR result of bikes, the points marked by the ▲ means the locations where people felt stressed

To explore further, we conducted a spatial analysis to investigate whether the association might vary depending on the location of the site. The GWR results are shown in Fig. 4, the red spots with the higher coefficient on the map indicate where participants may have higher physiological stress responses influenced by the predictors. It shows that locations with a high coefficient between physiological stress response and human crowds are found near roads in the blue space, narrow streets and squares in the commercial space and city centre in the transit space, and at the entrance to the green space. Furthermore, the high coefficient between the physiological stress response and the number of sitting facilities can be found in several roadside areas and squares in the commercial space.

Moreover, the result of the GWR also shows that the number of bicycles was associated with higher levels of stress response in the commercial space where the overwhelming number of parked bicycles are parked, and areas nearby intersections in the transit space. Regarding the association between motor vehicles and physiological stress response, we found that the number of vehicles was strongly associated with a high level of change score in the internal network of the commercial space. The results suggest that parked vehicles and bicycles in pedestrian areas with limited walking space may increase the physiological stress response.

As for the psychological responses of the participants collected through the eDiary app, we found that most of the reported stressful events occurred in the transit space near the city centre and the roadside commercial space, where the large human crowds, parked bicycles and vehicles coexist. Compared to subjective surveys, sensor-based measurement and GWR analysis provide a new quantitative method to investigate the impact of crowding on changes in human physiological stress response by location, with respect to different urban elements.

## Discussion

In this study, a new method was applied to measure personal exposure to overcrowding at street level and examined the association between urban overcrowding (human crowds, sitting facilities, bikes and motor vehicles) and human physiological stress responses through continuous personal tracking in different urban settings (blue space, transit space, green space, commercial space). This study confirmed the possibility of integrating wearable cameras, GPS devices and health trackers to statistically and spatially analyse the association between overcrowding and human stress at the individual level. Finally, this study suggests that more attention should be paid to the overwhelming number of people, parked bicycles and parked vehicles in pedestrian areas.

In this study, the number of people in the close distance and personal space can be regarded as a violation of the observer’s comfortable space in a commercial area. The result of the GLMM shows that close exposure to human crowds in a commercial space and transit space may increase people’s physiological stress response to overcrowding, but not in green space and blue space. The possible explanation is that the context may play the role of “buffering” to reduce the impact of overcrowding. As explained in previous studies, urban nature has therapeutic effects on humans’ stress [80, 81]. Besides, this study also finds the positive correlation between bikes and human stress response in the commercial space and blue space. Researchers suggest using a context-sensitive approach to enhance bike-friendly design and improve urban management of parked bikes [82, 83] and have suggested strategies for creating car-free zones [84] to improve urban livability and human health.

The GWR analysis facilitated the exploration of locations where people may experience physiological stress response caused by specific urban elements, which provides an opportunity to reflect on our design of urban space. For example, previous work has shown that reasonable amenities can encourage people to engage in staying activities in POS – essential to forming the local liveliness [85, 86]. These staying activities include standing, sitting, lying, talking, eating and drinking, reading, window shopping, smoking, vending, playing games, and listening to musicians [87]. However, we may need to reconsider whether roadside benches are frequently used, as benches close to traffic may not reduce people’s physiological stress response. Also, while sitting areas are welcome in cities, dining tables and chairs in busy pedestrian areas may increase people’s stress if there is insufficient space to walk. More empirical research can be done in future studies to address these considerations.

This study also finds it hard to explain the difference between objective measurement and subjective feedback, as the possible reasons are the small sample size and limited knowledge of psychology. However, it is worth discussing this issue in the next step for a comprehensive understanding of the impact of urban features on human health. We suggest using a pre-survey before the sensor-based measurement and follow-up studies, such as interviews, to assess participants’ perceptions of urban features and help interpret the results.

In recent years, researchers have explored urban design approaches to improve the design quality of the built environment to provide citizens with healthy environments [88, 89]. This paper focuses on three urban elements (sitting facilities, vehicles and bikes) and suggests that future studies should investigate more urban elements associated with overcrowding and social crowds, such as recreational facilities, exhibitions and street performances. Although urban planning in most European cities has evolved from car-oriented to pedestrian-oriented development, the “old” design in small-scale public open spaces needs renewal to benefit human health. Although  in many countries relevant stakeholders in urban governance have published design guidelines to improve street quality, current literature on how to make this transition remains scarce.

The main weakness of this study was the small number of participants, mainly from universities, which limits the generalisability of our findings. This is a common limitation for studies using personal sensors [68]. Future research is needed to confirm our work by recruiting a larger number of participants and to study how distinctive groups of people (e.g., age, gender, education, income) respond differently to the overcrowding in the same environment, with a particular focus on vulnerable groups of people (e.g., children and the elderly).

Another limitation relates to the geography and urban environment. Previous studies have explained that cultural factors and socio-economic differences are important confounding variables that may lead to different perceptions of overcrowding [90, 91]. More importantly, when replicating the experiment in different cities, researchers should consider the regional characteristics and local contexts rather than simply transferring known characteristics from other cities. For example, we suggest taking into account the height of buildings and the width of streets when discussing overcrowding in high-density cities.

The investigators also encountered problems regarding the sensors during the data collection. First, the connection between the sensors and the “eDiary” smartphone app was not stable at times, which may have led to data missing and failure of extracting features from data. Second, the battery of the cameras and phones unexpectedly ran out of power in the cold weather. Third, since the “Zephyr Bioharness 3” chest belt was designed to be worn around the chest and touching the skin, it was inconvenient for participants to put on and take off in the urban environment and difficult for investigators to check and restart sensors once connection was lost. We also found that the design of the chest belt is not friendly for female participants with smaller body sizes. Future studies could carefully address these technical issues to obtain high quality data. Besides, we suggest exploring other physiological data from E4, such as heart rate, and electrocardiogram from the chest belt, to gain a more comprehensive understanding of human stress.

This paper provides new evidence for integrating wearable cameras and wearable sensors to achieve individual-level objective measurements in urban environments [57]. However, cameras can capture images of cars from a personal perspective but cannot evaluate the overall crowdedness of traffic. Therefore, our findings cannot be extrapolated to determine specific stressors. Furthermore, although this paper categorised people into four distance zones based on their position and area in the imagery, the threshold value of distance zones is generated from this sample, which may not be the most appropriate threshold value in different conditions. In future studies, we suggest using a more advanced algorithm to find the optimal threshold value and detect the direction of human crowds.

In terms of analysis, we attempted to use statistical methods to explore the potential of fused data. In this study, we chose specific categories of urban elements and used the total number of objects detected from the images, resulting in sparse data where most of the values of the independent variables are zero and a high correlation between two or more independent variables. This also results in a very low R square in the GLMM analysis. Future studies can use a large data sample and regularisation methods to reduce the impact of highly correlated independent variables on the model. We also found that the residuals from the GWR model were over-predicted in some locations, so we recommend that future research should explore advanced models.

Lastly, this paper did not control for the possible confounding effects of other environmental and social factors, such as traffic noise, people’s yelling from nearby recreational facilities, and protests against coronavirus vaccination on the streets, which may have increased people’s stress responses. The severity of COVID-19 outbreak in Salzburg during the fieldwork period, and the announced lockdown might have effects on people’s responses to the overcrowding.

## Conclusion

In this study, Salzburg was used as a case study to test the feasibility of using wearable cameras to measure street-level overcrowding, and to provide a workflow for statistically and spatially investigating the relationship between overcrowding and human physiological stress response at the individual level. We believe that this work provides a new method for studying personal space and overcrowding and their impact on human health, which can inspire urban planners and policy makers to consider negative environments from a human perspective.

## Supplementary Information

**Additional file 1** **Figure S1.** the standardized residual from GWR model.

**Additional file 2**: **Table S1.** comparation between GWR model and Ordinary least-squares (OLS) model.

## Data Availability

The data has been used is confidential.

## Footnotes

1 The four types of “distance zones” around humans identified in current literature are intimate (0–18 in), personal (18in–4 ft.), social (4–12 ft.), and public (12 + ft).

2 https://www.empatica.com/en-int/.

3 https://support.microsoft.com/en-us/topic/what-can-i-still-do-with-my-microsoft-band-a2a59355-5be0-3441-9fff-4dc27bcbafb5.

4 https://www.fitbit.com/global/dk/home.
