# Supplementary figures and images for: Assessing the association between overcrowding and human physiological stress response in different urban contexts: a case study in Salzburg, Austria
Source: Int J Health Geogr. 2023 Jun 21;22:15. doi: 10.1186/s12942-023-00334-7 (PMC10286433; doi:10.1186/s12942-023-00334-7)

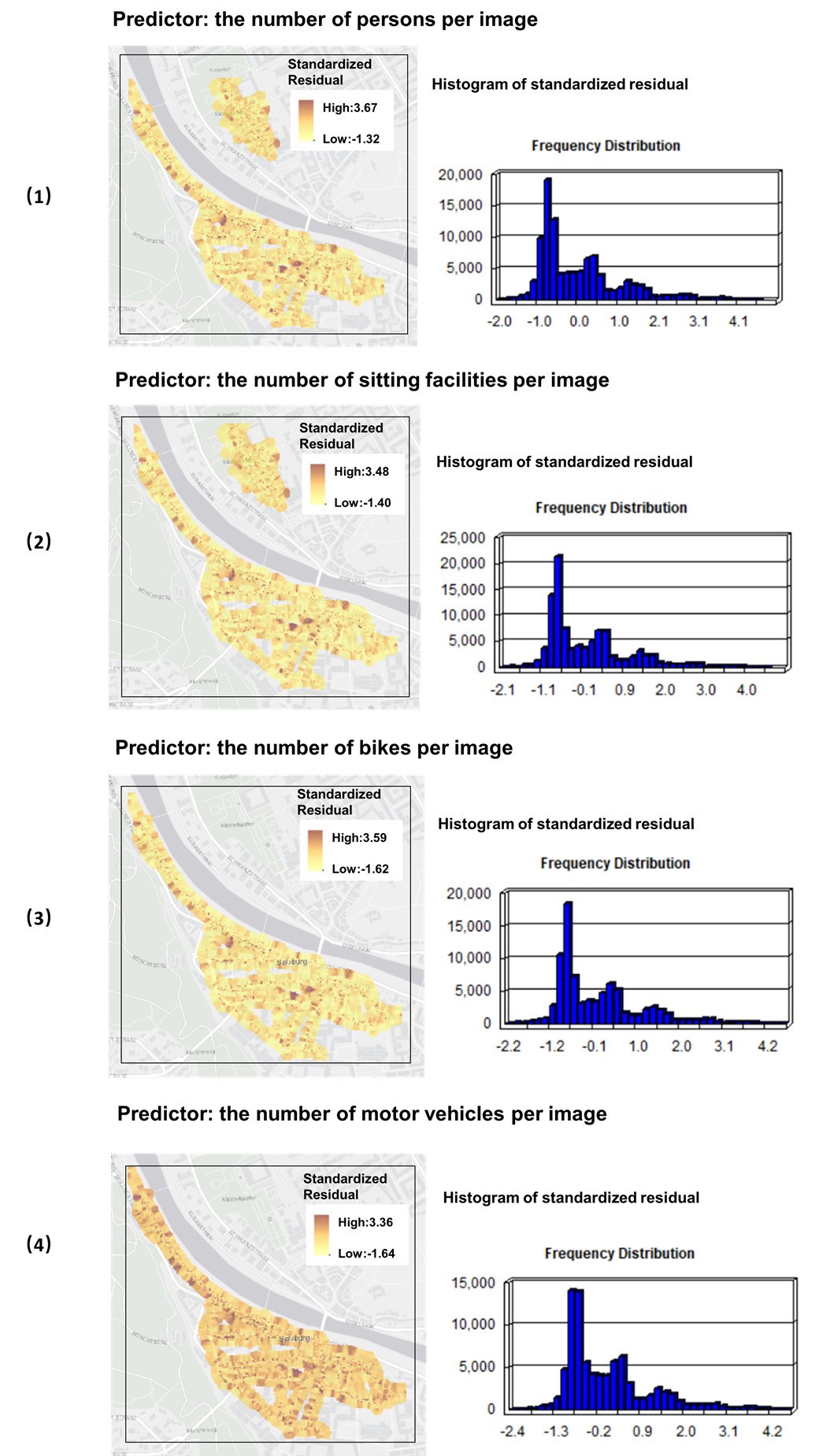

Supplement: Supplementary file 1 — Additional file 1 Figure S1. the standardized residual from GWR model. [file 12942_2023_334_MOESM1_ESM.jpg]
