# Supplementary material for: Assessing the association between overcrowding and human physiological stress response in different urban contexts: a case study in Salzburg, Austria
Source: Int J Health Geogr. 2023 Jun 21;22:15. doi: 10.1186/s12942-023-00334-7 (PMC10286433; doi:10.1186/s12942-023-00334-7)
Supplement: Supplementary file 2 — Additional file 2: Table S1. comparation between GWR model and Ordinary least-squares (OLS) model. [file 12942_2023_334_MOESM2_ESM.docx]

**Appendix table S1**

| **Predictor: human crowds; Response: change score** | | | |
| --- | --- | --- | --- |
|  | AICc | R2 | R2Adjusted |
| OLS | 840164.8659 | 0.022 | 0.000 |
| GWR | 833887.91 | 0.07 | 0.06 |
| Predictor: sitting facility; Response: change score | | | |
|  | AICc | R2 | R2Adjusted |
| OLS | 840203.6677 | 0.02 | 0.000 |
| GWR | 835164.82 | 0.05 | 0.05 |
| Predictor: bikes; Response: change score | | | |
|  | AICc | R2 | R2Adjusted |
| OLS | 840203.77 | 0.02 | 0.000 |
| GWR | 698566.79 | 0.06 | 0.057 |
| Predictor: Motor vehicles; Response: change score | | | |
|  | AICc | R2 | R2Adjusted |
| OLS | 840157.9447 | 0.02 | 0.000 |
| GWR | 696504.49 | 0.09 | 0.08 |
